# Crystal structure of lead(II) tartrate: a redetermination

**DOI:** 10.1107/S2056989014027376

**Published:** 2015-01-01

**Authors:** Matthias Weil

**Affiliations:** a Institute for Chemical Technologies and Analytics, Division of Structural Chemistry, Vienna University of Technology, Getreidemarkt 9/164-SC, A-1060 Vienna, Austria

**Keywords:** crystal structure, lead tartrate, gel growth, redetermination, O—H⋯O hydrogen bonds

## Abstract

Single crystals of poly[μ_4_-tartrato-κ^6^ *O* ^1^,*O* ^3^:*O* ^1′^:*O* ^2^,*O* ^4^:*O* ^4′^-lead], [Pb(C_4_H_4_O_6_)]_*n*_, were grown in a gel medium. In comparison with the previous structure determination of this compound from laboratory powder X-ray diffraction data [De Ridder *et al.* (2002 ▸). *Acta Cryst.* C**58**, m596–m598], the redetermination on the basis of single-crystal data reveals the absolute structure, all atoms with anisotropic displacement parameters and a much higher accuracy in terms of bond lengths and angles. It could be shown that a different space group or incorporation of water as reported for similarly gel-grown lead tartrate crystals is incorrect. In the structure, each Pb^2+^ cation is bonded to eight O atoms of five tartrate anions, while each tartrate anion links four Pb^2+^ cations. The resulting three-dimensional framework is stabilized by O—H⋯O hydrogen bonds between the OH groups of one tartrate anion and the carboxyl­ate O atoms of adjacent anions.

## Abstract 2

The redetermination of the crystal structure of lead tartrate from crystals grown in a gel medium confirmed the previous powder X-ray diffraction study in the space group *P*2_1_2_1_2_1_ with higher precision. Contradictions in the literature regarding space group and water content could be clarified.

## Chemical context   

Crystal growth in gels (Henisch, 1970 ▸) is a convenient method to obtain single crystals of high quality from compounds with rather low solubility products. Therefore gel growth was the method of choice for single-crystal growth of the low-soluble fluoro­phosphate BaPO_3_F. This compound is inter­esting insofar as the polycrystalline material (prepared by fast precipitation) has ortho­rhom­bic symmetry whereas single crystals grown slowly in a gel have monoclinic symmetry. Both the ortho­rhom­bic and monoclinic BaPO_3_F phases belong to the same order–disorder (OD) family and can be derived from the baryte (BaSO_4_) structure type by replacing the SO_4_^2−^ anions with isoelectronic PO_3_F^2−^ anions in two orientations (Stöger *et al.*, 2013 ▸). The same baryte-type structure has been reported for PbPO_3_F on the basis of similar lattice parameters and systematic absences of reflections (Walford, 1967 ▸). However, structural details were not determined at that time. In analogy with the barium compound, it was intended to grow crystals of lead fluoro­phosphate in a gel medium. In order to take into account the somewhat lower solubility of PbPO_3_F in comparison with BaPO_3_F (Lange, 1929 ▸), crystal-growth experiments were performed with lead salts in ammoniacal tartrate solutions to produce a soluble, poorly dissociated lead tartrate complex which lowers the concentration of Pb^2+^ to such an extent that its direct precipitation is prevented. In fact, after some days colourless single crystals appeared in the gel medium that, on the basis of unit-cell determinations, turned out to be lead tartrate, [Pb(C_4_H_4_O_6_)]. The structure of this compound was originally solved and refined from laboratory X-ray powder diffraction data in space group *P*2_1_2_1_2_1_ (De Ridder *et al.*, 2002 ▸). However, some years later it was reported that gel-grown lead tartrate crystallizes as a dihydrate (Lillybai & Rahimkutty, 2010 ▸) or in a different space group (*Pna*2_1_; Labutina *et al.*, 2011 ▸). Motivated by these disagreements, it was decided to re-investigate the crystal structure of gel-grown lead tartrate on the basis of single-crystal diffraction data for an unambiguous determination of the space group and the composition, and to obtain more precise results compared to the powder refinement.
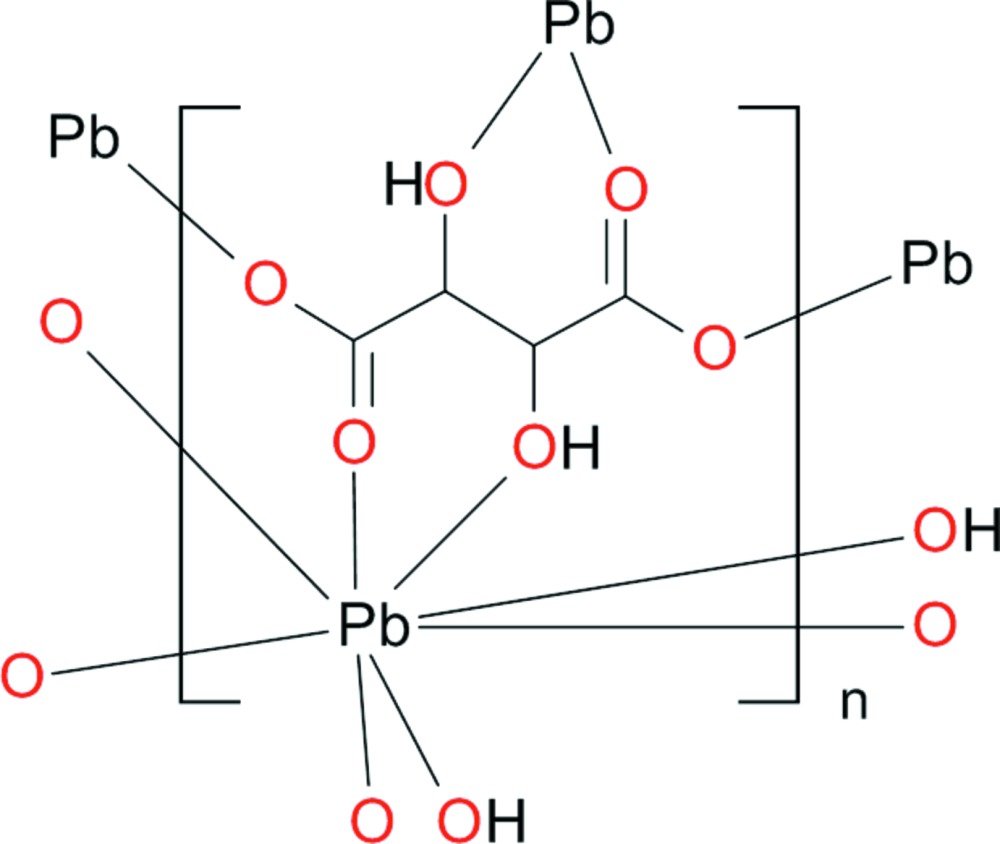


## Structural commentary   

The present study confirms in principle the results of the previous powder X-ray diffraction study and reveals the determination of the absolute structure (Flack parameter 0.003 (7); Flack, 1983 ▸) and all non-H atoms refined with anisotropic displacement parameters. In comparison with the powder study, the higher precision and accuracy of the present model is, for example, reflected by the notable differences in the Pb—O bond lengths determined in the two studies (Table 1 ▸). An important result of the present study is that neither a different space group nor a different content in terms of an incorporation of water into the structure could be found on the basis of the single-crystal data.

**Figure 1:**
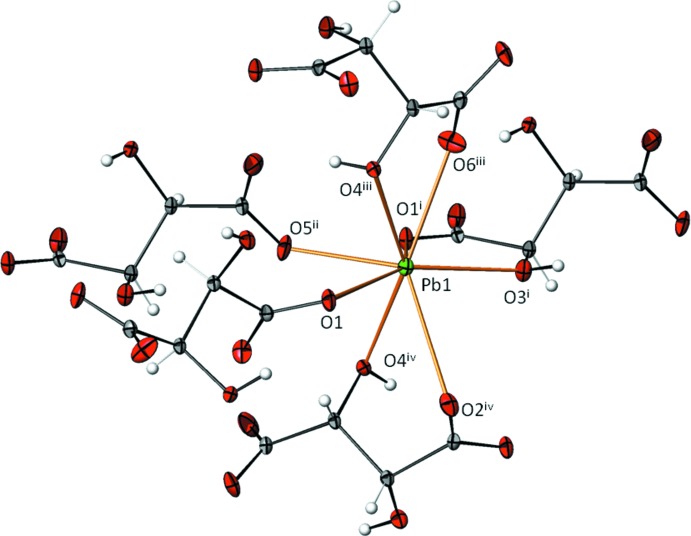
Coordination environment of the Pb^2+^ cation in the title compound, with atom labelling (for symmetry codes refer to Table 1 ▸). Displacement ellipsoids are drawn at the 50% probability level.

The Pb^2+^ cation has a coordination number of eight considering a cut-off value of 3 Å for the ligating oxygen atoms. The coordination polyhedron is considerably distorted (Fig. 1 ▸), with Pb—O distances in the range 2.472 (2)–3.004 (2) Å (Table 1 ▸). The resulting bond-valence sum (Brown, 2002 ▸) of 1.75 valence units, using the parameters of Krivovichev & Brown (2001 ▸) for the Pb—O bonds, is reasonably close to the expected value of 2.0 valence units. Bond lengths and angles within the tartrate anion are in normal ranges.

## Packing features   

**Figure 2:**
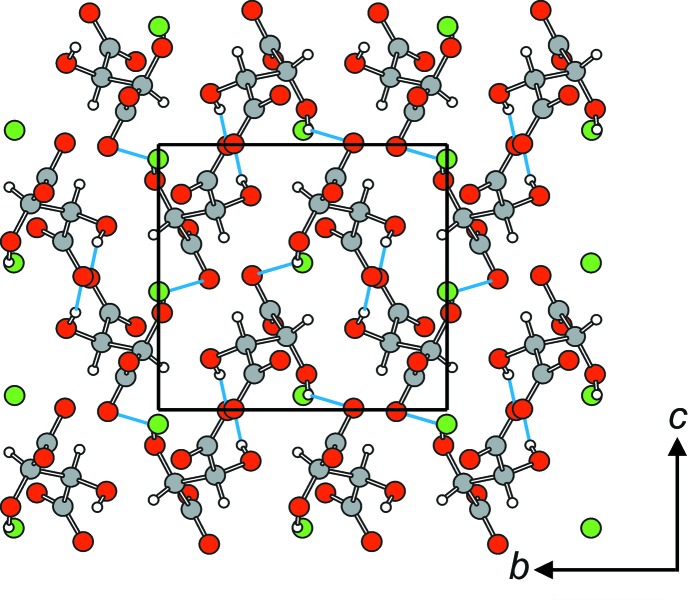
The crystal packing of the title compound in projection along [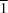
00]. Only complete tartrate anions are shown. O—H⋯O hydrogen bonds are shown in blue (see Table 2 ▸ for details). Pb—O bonds have been omitted for clarity. Colour code: Pb green, C grey, O red, H white.

In the crystal structure, the Pb^2+^ cations are arranged in hexa­gonally packed rows extending parallel to [100] (Fig. 2 ▸). Each Pb^2+^ cation is bonded to five tartrate anions (three chelating and two in a monodentate fashion, Fig. 1 ▸) while each tartrate anion links four Pb^2+^ cations, leading to a three-dimensional framework. O—H⋯O hydrogen bonds (Table 2 ▸) between the hy­droxy groups of one tartrate anion and the carboxyl­ate O atoms of adjacent tartrate anions stabilize this arrangement. Since no solvent-accessible voids were observed in the crystal structure, an incorporation of water mol­ecules as reported by Lillibay & Rahimkutty (2010) is impossible.

## Database survey   

Tartaric acid and its salts or coordination compounds have been structurally examined in great detail. The current release of the CSD (Version 5.35 with all updates; Groom & Allen, 2014 ▸) revealed 644 entries, including the pure acid, co-crystals, compounds with the hydrogen tartrate anion and compounds with the tartrate anion.

## Synthesis and crystallization   

Commercially available gelatin was dissolved in hot water. The solution (50 ml) was cooled to about 300 K and 300 mg of (NH_4_)_2_(PO_3_F)(H_2_O), prepared according to Schülke & Kayser (1991 ▸), were dissolved in the still liquid solution that was filled in a large test tube. After initiation of gelling, a second neutral gel layer was put on top of the first gel layer. After the neutral gel had set, an aqueous solution consisting of Pb(NO_3_)_2_ (30 mg) and sodium potassium tartrate (250 mg) was poured over the second gel layer. After three weeks, colourless single crystals of lead(II) tartrate, mostly with a block-like form, could be isolated. PbPO_3_F in the form of polycrystalline material was also present in the reaction mixture as revealed by powder X-ray diffraction measurements.

## Refinement   

Crystal data, data collection and structure refinement details are summarized in Table 3 ▸. Atom labelling and starting coordinates for the refinement were taken from the previous powder diffraction study (De Ridder *et al.*, 2002 ▸). H atoms bonded to C atoms were placed in calculated positions and refined as riding atoms, with C—H = 0.98 Å and with *U*_iso_(H) = 1.2*U*_eq_(C). Hydroxyl H atoms were found from difference Fourier maps and refined with an O—H distance restraint of 0.85 (1) Å and with *U*_iso_(H) = 1.2*U*_eq_(O). The highest and lowest remaining electron densities are found 0.59 and 0.49 Å, respectively, from the Pb atom and are caused by truncation effects. No other electron densities attributable to additional atoms could be found, ruling out an incorporation of water mol­ecules. Refinements in space group *Pna*2_1_ as suggested by Labutina *et al.* (2011 ▸) led to unreasonable models.

## Supplementary Material

Crystal structure: contains datablock(s) I, general. DOI: 10.1107/S2056989014027376/su5041sup1.cif

Structure factors: contains datablock(s) I. DOI: 10.1107/S2056989014027376/su5041Isup2.hkl

CCDC reference: 1039408

Additional supporting information:  crystallographic information; 3D view; checkCIF report

## Figures and Tables

**Table 1:** Comparison of the PbO bond lengths () in the current and the previous (De Ridder *et al.*, 2002 ▸) refinements of lead tartrate For the previous refinement: *a* = 7.99482(3), *b* = 8.84525(4), *c* = 8.35318(4).

Bond	current refinement	previous refinement
PbO1^i^	2.472(2)	2.859(12)
PbO5^ii^	2.482(2)	2.398(11)
PbO6^iii^	2.594(2)	2.575(12)
PbO3^i^	2.5972(17)	2.637(9)
PbO4^iv^	2.6878(19)	2.649(11)
PbO4^iii^	2.7866(19)	2.847(12)
PbO2^iv^	2.935(2)	2.975(13)
PbO1	3.004(2)	2.754(12)

Symmetry codes: (i) *x* 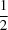, *y*, *z* 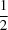; (ii) *x*+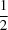, *y*, *z* 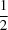; (iii) *x*, *y* 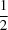, *z*+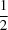; (iv) *x* 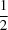, *y*+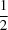, *z*.

**Table 2:** Hydrogen-bond geometry (, )

*D*H*A*	*D*H	H*A*	*D* *A*	*D*H*A*
O3H3*O*O2^i^	0.84(1)	2.02(4)	2.646(3)	131(5)
O4H4*O*O6^ii^	0.85(1)	1.79(1)	2.618(3)	169(4)

Symmetry codes: (i) 
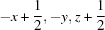
; (ii) 
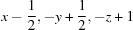
.

**Table 3:** Experimental details

Crystal data	
Chemical formula	[Pb(C_4_H_4_O_6_)]
*M*_r_	355.26
Crystal system, space group	Orthorhombic, *P*2_1_2_1_2_1_
Temperature (K)	296
*a*, *b*, *c* ()	7.9890(2), 8.8411(3), 8.3434(2)
*V* (^3^)	589.31(3)
*Z*	4
Radiation type	Mo *K*
(mm^1^)	28.61
Crystal size (mm)	0.15 0.15 0.09

Data collection	
Diffractometer	Bruker APEXII CCD
Absorption correction	Multi-scan (*SADABS*; Bruker, 2013 ▸)
*T*_min_, *T*_max_	0.099, 0.183
No. of measured, independent and observed [*I* > 2(*I*)] reflections	81742, 4512, 3836
*R*_int_	0.046
(sin /)_max_ (^1^)	0.971

Refinement	
*R*[*F* ^2^ > 2(*F* ^2^)], *wR*(*F* ^2^), *S*	0.021, 0.042, 1.06
No. of reflections	4512
No. of parameters	108
No. of restraints	2
H-atom treatment	H atoms treated by a mixture of independent and constrained refinement
_max_, _min_ (e ^3^)	3.20, 2.26
Absolute structure	Flack (1983 ▸), 1959 Friedel pairs
Absolute structure parameter	0.003(7)

Computer programs: *APEX2* and *SAINT-Plus* (Bruker, 2013 ▸), *SHELXS97* and *SHELXL97* (Sheldrick, 2008 ▸), *ATOMS* (Dowty, 2006 ▸) and *publCIF* (Westrip, 2010 ▸).

## supplementary crystallographic information

### Crystal data

**Table:**

[Pb(C_4_H_4_O_6_)]	*F*(000) = 632
*M**_r_* = 355.26	*D*_x_ = 4.004 Mg m^−^^3^
Orthorhombic, *P*2_1_2_1_2_1_	Mo *K*α radiation, λ = 0.71073 Å
Hall symbol: P 2ac 2ab	Cell parameters from 9854 reflections
*a* = 7.9890 (2) Å	θ = 3.4–40.9°
*b* = 8.8411 (3) Å	µ = 28.61 mm^−^^1^
*c* = 8.3434 (2) Å	*T* = 296 K
*V* = 589.31 (3) Å^3^	Block, colourless
*Z* = 4	0.15 × 0.15 × 0.09 mm

### Data collection

**Table:**

Bruker APEXII CCD diffractometer	4512 independent reflections
Radiation source: fine-focus sealed tube	3836 reflections with *I* > 2σ(*I*)
Graphite monochromator	*R*_int_ = 0.046
ω– and φ–scans	θ_max_ = 43.7°, θ_min_ = 3.4°
Absorption correction: multi-scan (*SADABS*; Bruker, 2013)	*h* = −15→15
*T*_min_ = 0.099, *T*_max_ = 0.183	*k* = −17→17
81742 measured reflections	*l* = −16→16

### Refinement

**Table:**

Refinement on *F*^2^	Secondary atom site location: difference Fourier map
Least-squares matrix: full	Hydrogen site location: inferred from neighbouring sites
*R*[*F*^2^ > 2σ(*F*^2^)] = 0.021	H atoms treated by a mixture of independent and constrained refinement
*wR*(*F*^2^) = 0.042	*w* = 1/[σ^2^(*F*_o_^2^) + (0.0155*P*)^2^ + 1.1508*P*] where *P* = (*F*_o_^2^ + 2*F*_c_^2^)/3
*S* = 1.06	(Δ/σ)_max_ = 0.007
4512 reflections	Δρ_max_ = 3.20 e Å^−^^3^
108 parameters	Δρ_min_ = −2.26 e Å^−^^3^
2 restraints	Absolute structure: Flack (1983), 1959 Friedel pairs
Primary atom site location: structure-invariant direct methods	Absolute structure parameter: −0.003 (7)

### Special details

**Table:**

Geometry. All e.s.d.'s (except the e.s.d. in the dihedral angle between two l.s. planes) are estimated using the full covariance matrix. The cell e.s.d.'s are taken into account individually in the estimation of e.s.d.'s in distances, angles and torsion angles; correlations between e.s.d.'s in cell parameters are only used when they are defined by crystal symmetry. An approximate (isotropic) treatment of cell e.s.d.'s is used for estimating e.s.d.'s involving l.s. planes.
Refinement. Refinement of *F*^2^ against ALL reflections. The weighted *R*-factor *wR* and goodness of fit *S* are based on *F*^2^, conventional *R*-factors *R* are based on *F*, with *F* set to zero for negative *F*^2^. The threshold expression of *F*^2^ > σ(*F*^2^) is used only for calculating *R*-factors(gt) *etc*. and is not relevant to the choice of reflections for refinement. *R*-factors based on *F*^2^ are statistically about twice as large as those based on *F*, and *R*- factors based on ALL data will be even larger.

### Fractional atomic coordinates and isotropic or equivalent isotropic displacement parameters (Å^2^)

**Table:**

	*x*	*y*	*z*	*U*_iso_*/*U*_eq_	
Pb1	−0.342448 (9)	0.001349 (17)	−0.054104 (9)	0.01396 (2)	
O1	−0.0806 (2)	0.1027 (2)	0.1809 (3)	0.0159 (3)	
O2	0.1001 (3)	0.1758 (3)	−0.0066 (2)	0.0180 (4)	
O3	0.1564 (2)	−0.0126 (3)	0.3659 (2)	0.0147 (3)	
O4	0.2220 (2)	0.3195 (2)	0.3069 (2)	0.0123 (3)	
O5	0.5788 (3)	0.0785 (3)	0.3132 (3)	0.0215 (4)	
O6	0.4746 (3)	0.2388 (3)	0.4968 (3)	0.0244 (5)	
C1	0.0635 (3)	0.1138 (3)	0.1249 (3)	0.0103 (3)	
C2	0.2130 (3)	0.0566 (3)	0.2235 (3)	0.0100 (3)	
H2	0.2764	−0.0171	0.1601	0.012*	
C3	0.3222 (3)	0.1966 (3)	0.2538 (3)	0.0101 (3)	
H3	0.3686	0.2263	0.1497	0.012*	
C4	0.4706 (3)	0.1686 (3)	0.3642 (3)	0.0128 (4)	
H3O	0.224 (4)	−0.019 (6)	0.443 (4)	0.029 (12)*	
H4O	0.144 (4)	0.288 (5)	0.367 (4)	0.015 (10)*	

### Atomic displacement parameters (Å^2^)

**Table:**

	*U*^11^	*U*^22^	*U*^33^	*U*^12^	*U*^13^	*U*^23^
Pb1	0.01063 (3)	0.01446 (3)	0.01679 (3)	0.00067 (7)	−0.00006 (2)	0.00120 (7)
O1	0.0081 (6)	0.0230 (9)	0.0167 (8)	0.0000 (6)	−0.0005 (6)	0.0032 (7)
O2	0.0155 (8)	0.0274 (10)	0.0111 (7)	0.0026 (8)	−0.0009 (6)	0.0057 (7)
O3	0.0116 (5)	0.0192 (9)	0.0134 (6)	0.0001 (8)	0.0009 (5)	0.0072 (7)
O4	0.0092 (6)	0.0113 (7)	0.0165 (8)	0.0011 (5)	0.0001 (6)	0.0004 (6)
O5	0.0100 (7)	0.0277 (11)	0.0267 (11)	0.0073 (7)	−0.0009 (7)	−0.0005 (8)
O6	0.0252 (10)	0.0242 (10)	0.0237 (10)	0.0083 (9)	−0.0156 (9)	−0.0090 (8)
C1	0.0081 (8)	0.0120 (8)	0.0108 (8)	−0.0009 (6)	−0.0017 (6)	−0.0006 (6)
C2	0.0081 (7)	0.0121 (8)	0.0098 (8)	0.0003 (7)	0.0004 (6)	0.0011 (6)
C3	0.0067 (8)	0.0129 (8)	0.0107 (8)	0.0005 (6)	−0.0001 (6)	0.0011 (6)
C4	0.0075 (7)	0.0137 (9)	0.0172 (10)	−0.0003 (7)	−0.0017 (7)	0.0003 (7)

### Geometric parameters (Å, º)

**Table:**

Pb1—O1^i^	2.472 (2)	O3—H3O	0.843 (10)
Pb1—O5^ii^	2.482 (2)	O4—C3	1.420 (3)
Pb1—O6^iii^	2.594 (2)	O4—H4O	0.845 (10)
Pb1—O3^i^	2.5972 (17)	O5—C4	1.250 (3)
Pb1—O4^iv^	2.6878 (19)	O6—C4	1.270 (3)
Pb1—O4^iii^	2.7866 (19)	C1—C2	1.536 (3)
Pb1—O2^iv^	2.935 (2)	C2—C3	1.535 (3)
Pb1—O1	3.004 (2)	C2—H2	0.9800
O1—C1	1.246 (3)	C3—C4	1.522 (3)
O2—C1	1.261 (3)	C3—H3	0.9800
O3—C2	1.411 (3)		
			
O1^i^—Pb1—O5^ii^	72.93 (7)	C1—O1—Pb1^v^	126.48 (16)
O1^i^—Pb1—O6^iii^	74.36 (7)	C2—O3—Pb1^v^	120.65 (14)
O5^ii^—Pb1—O6^iii^	99.96 (9)	C2—O3—H3O	118 (3)
O1^i^—Pb1—O3^i^	62.88 (6)	Pb1^v^—O3—H3O	115 (3)
O5^ii^—Pb1—O3^i^	135.70 (7)	C3—O4—Pb1^vi^	108.28 (13)
O6^iii^—Pb1—O3^i^	71.85 (8)	C3—O4—H4O	111 (3)
O1^i^—Pb1—O4^iv^	64.23 (6)	Pb1^vi^—O4—H4O	121 (3)
O5^ii^—Pb1—O4^iv^	69.82 (7)	C4—O5—Pb1^vii^	128.07 (19)
O6^iii^—Pb1—O4^iv^	138.58 (7)	C4—O6—Pb1^viii^	125.98 (18)
O3^i^—Pb1—O4^iv^	87.75 (6)	O1—C1—O2	125.1 (2)
O1^i^—Pb1—O4^iii^	122.21 (6)	O1—C1—C2	119.4 (2)
O5^ii^—Pb1—O4^iii^	82.70 (7)	O2—C1—C2	115.4 (2)
O6^iii^—Pb1—O4^iii^	59.20 (6)	O3—C2—C3	113.16 (19)
O3^i^—Pb1—O4^iii^	123.07 (6)	O3—C2—C1	110.14 (19)
O4^iv^—Pb1—O4^iii^	148.738 (15)	C3—C2—C1	105.30 (18)
O1^i^—Pb1—O2^iv^	118.51 (7)	O3—C2—H2	109.4
O5^ii^—Pb1—O2^iv^	119.10 (7)	C3—C2—H2	109.4
O6^iii^—Pb1—O2^iv^	140.78 (8)	C1—C2—H2	109.4
O3^i^—Pb1—O2^iv^	81.74 (7)	O4—C3—C4	111.99 (19)
O4^iv^—Pb1—O2^iv^	65.93 (6)	O4—C3—C2	110.38 (18)
O4^iii^—Pb1—O2^iv^	119.18 (6)	C4—C3—C2	114.25 (19)
O1^i^—Pb1—O1	150.22 (4)	O4—C3—H3	106.6
O5^ii^—Pb1—O1	77.58 (7)	C4—C3—H3	106.6
O6^iii^—Pb1—O1	115.47 (6)	C2—C3—H3	106.6
O3^i^—Pb1—O1	145.98 (6)	O5—C4—O6	126.2 (3)
O4^iv^—Pb1—O1	101.71 (6)	O5—C4—C3	115.9 (2)
O4^iii^—Pb1—O1	56.54 (5)	O6—C4—C3	117.9 (2)
O2^iv^—Pb1—O1	72.91 (6)		

Symmetry codes: (i) −*x*−1/2, −*y*, *z*−1/2; (ii) −*x*+1/2, −*y*, *z*−1/2; (iii) −*x*, *y*−1/2, −*z*+1/2; (iv) *x*−1/2, −*y*+1/2, −*z*; (v) −*x*−1/2, −*y*, *z*+1/2; (vi) *x*+1/2, −*y*+1/2, −*z*; (vii) −*x*+1/2, −*y*, *z*+1/2; (viii) −*x*, *y*+1/2, −*z*+1/2.

### Hydrogen-bond geometry (Å, º)

**Table:**

*D*—H···*A*	*D*—H	H···*A*	*D*···*A*	*D*—H···*A*
O3—H3*O*···O2^vii^	0.84 (1)	2.02 (4)	2.646 (3)	131 (5)
O4—H4*O*···O6^ix^	0.85 (1)	1.79 (1)	2.618 (3)	169 (4)

Symmetry codes: (vii) −*x*+1/2, −*y*, *z*+1/2; (ix) *x*−1/2, −*y*+1/2, −*z*+1.
